# Inference of Structure Ensembles of Flexible Biomolecules from Sparse, Averaged Data

**DOI:** 10.1371/journal.pone.0079439

**Published:** 2013-11-07

**Authors:** Simon Olsson, Jes Frellsen, Wouter Boomsma, Kanti V. Mardia, Thomas Hamelryck

**Affiliations:** 1 Bioinformatics Centre, Department of Biology, Faculty of Science, University of Copenhagen, Copenhagen, Denmark; 2 Structural Biology and NMR Laboratory, Department of Biology, Faculty of Science, University of Copenhagen, Copenhagen, Denmark; 3 Department of Statistics, School of Mathematics, University of Leeds, Leeds, United Kingdom; Aberystwyth University, United Kingdom

## Abstract

We present the theoretical foundations of a general principle to infer structure ensembles of flexible biomolecules from spatially and temporally averaged data obtained in biophysical experiments. The central idea is to compute the Kullback-Leibler optimal modification of a given prior distribution 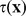 with respect to the experimental data and its uncertainty. This principle generalizes the successful inferential structure determination method and recently proposed maximum entropy methods. Tractability of the protocol is demonstrated through the analysis of simulated nuclear magnetic resonance spectroscopy data of a small peptide.

## Introduction

The rigorous analysis of experimental data probing the structure of biological macromolecules forms the foundation of many biophysical studies [1]. The sources of experimental data include nuclear magnetic resonance spectroscopy spectroscopy (NMR) and small-angle X-ray- and neutron scattering. This article addresses several issues which often make inference of biomolecular structure from such data particularly challenging. First, in these experiments, the time-scale of acquisition typically exceeds that of molecular fluctuations. Second, the samples studied are often near molar concentrations. Third, data is frequently incomplete, or even sparse, and subject to experimental noise. Consequently, data obtained from such techniques yield incomplete, noisy, spatially and temporally averaged information on the Boltzmann ensemble of the observed system. Thus, such data are ideally analyzed through models that take these properties into account. While this fact has long been recognized, the analysis of these types of data has revolved predominantly around *structure determination* – that is, fitting a single conformation to fulfill all derived geometrical restraints [2]. Such structure determination methods do not adequately handle sparse, noisy and averaged data. Here, we propose an alternative method which addresses these shortcomings.

Typically, structure determination from experimental data proceeds through hybrid energy minimization [3]. In this method, an energy function 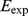 that brings in the experimental data is combined with an approximative physical forcefield 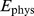. The term 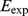 is typically a straight-forward combination of a forward- and an error-model. A forward-model relates a protein conformation to experimental data, whereas an error-model concerns experimental errors. Alternatively, a Bayesian formulation known as *inferential structure determination* (ISD) has been proposed, formulating structure determination in a rigorous probabilistic framework [4]. In ISD, a posterior distribution is constructed by combining a data likelihood with prior distributions on conformational and nuisance parameters. The likelihood and the prior concerning biomolecular structure correspond to 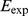 and 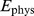, respectively. This Bayesian approach extends the common hybrid energy minimization by solving the important problems of choosing appropriate error-models, treating model-parameters coherently and performing inference through posterior sampling rather than minimization. However, by construction, these approaches assume that conformational variability can be represented through uncorrelated, homoscedastic fluctuations around one *average* structural representation. Consequently, the conformational heterogeneity present in the posterior distribution reflects the quality and completeness of the experimental data and the prior distributions, but not necessarily any physical fluctuations [5]. Despite this well-known limitation, the approximation tends to yield good results for well-folded proteins when conformational fluctuations are modest.

Early attempts to model ensemble NMR data involved averaging along molecular dynamics trajectories [6], [7]. In these protocols, a memory function specifies an averaging time-span which is used to obtain a time-averaged representation of the experimental data. While this approach displayed initial promise, the short timescales accessible through routine molecular dynamics limit its use [8]. An alternative approach, which involves explaining the data using an average of several conformations, emerged around the same time [9]. This approach has since shown to be more viable.

During the past two decades there has been an increasing interest in biomolecules that undergo significant conformational fluctuations, such as natively unfolded and partially unfolded proteins [10]. Consequently, there have been many efforts to overcome the limitations of structure determination procedures with respect to the flexibility of these molecular systems. Prevalently, conformational fluctuations are represented by finite *ensembles*: the data is explained by a weighed average of 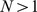 conformations, introduced above. In effect, this corresponds to discretizing the Boltzmann ensemble. Such discrete ensembles may be constructed in a multitude of ways, including database-derived explicit ensembles [11], [12], data-optimized explicit ensembles [13]–[17], fragment based ensemble construction [18]–[20] and multi-conformer refinement, molecular dynamics and Monte Carlo methods [8], [21]–[23] and maximum entropy methods [24]. Another important approach uses multiple replicas in the calculation of the hybrid energy used in restrained molecular simulations [8], [25], [26]. However, the discretization of the conformational ensemble is inherently problematic because determining the optimal ensemble size 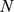, and its associated uncertainty, is difficult.

Restraining simulations using an average of multiple replicas is a sensible solution, as it was recently shown that multiple replica restrained simulations constitute the least biased method when the number of replicas goes to infinity in the absence of experimental noise [27]–[29]. However, a measurable bias is introduced when the number of replicas used is too small [28]. Since the use of large numbers of replicas may prove to be computationally intractable or impossible, the development of approaches which are independent of this discretization is highly desirable.

In this work, we approach the problem of modeling sparse, spatially and temporally averaged data through the principles of Bayesian statistics and information theory. Unlike the previous Bayesian efforts [4], [16], we explicitly take into account the experimental data as noisy, average quantities of an underlying heterogenous ensemble in continuous space. We derive a general posterior distribution from first principles which imposes the least necessary bias on our prior knowledge to fulfill the experimental data.

We outline a number of general, theoretical advances concerning biomolecular structure determination and restrained molecular simulations. To ensure a focused and concise presentation we limited the number of practical examples. However, one example given uses synthetic data of a small idealized peptide GB1 generated using the PROFASI forcefield at high temperature [30]. This choice allows us to carefully evaluate the theory presented by avoiding confounding variables. Finally, our findings are compared to existing methodology and is shown to generalize these.

## Results and Discussion

### A hierarchical model of spatially and temporally averaged restraints

Ultimately, our aim is to sample from the conditional probability distribution 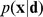, where 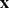 denotes a protein's conformation and 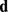 denotes spatially and temporally averaged, experimental data. The variable 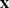 represents a positional microstate in atomic detail. Through a forward model 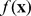 we can calculate a coarse-grained representation, 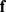, of a protein conformation 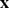. That is, our forward model is a mapping, 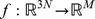, of the 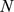 atoms of 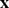 to an 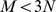-dimensional coarse grained representation, 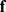. Conceptually, 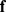 may be interpreted as the instantaneous 'experimental data' back-calculated from a positional micro-state, 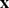. However, as 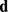 represents an averaged quantity we need to introduce a variable, 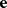, to represent an *ensemble average* of the simulated experimental data 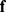. Consequently, our full posterior distribution becomes 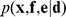.

We clarify the relation between 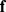, 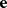 and 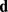 using the example we will present later on. In the case of nuclear Overhauser enhancement (NOE) data obtained from an NMR experiment [31], the coarse-grained variable 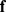 is a vector related to pairwise distances between atoms in a protein conformation 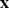. In one case, this is simply a vector of these distances. The variable 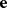 is an average of 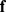 vectors from an ensemble of protein conformations. The experimental NOE data 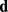 can be interpreted as a noisy observation of the vector of averages, 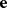. In general, there is no simple relationship between the vector 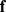 and the averaged vector 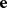, but a simple probabilistic model that relates them can be developed, as we discuss next.

**Figure 1:**
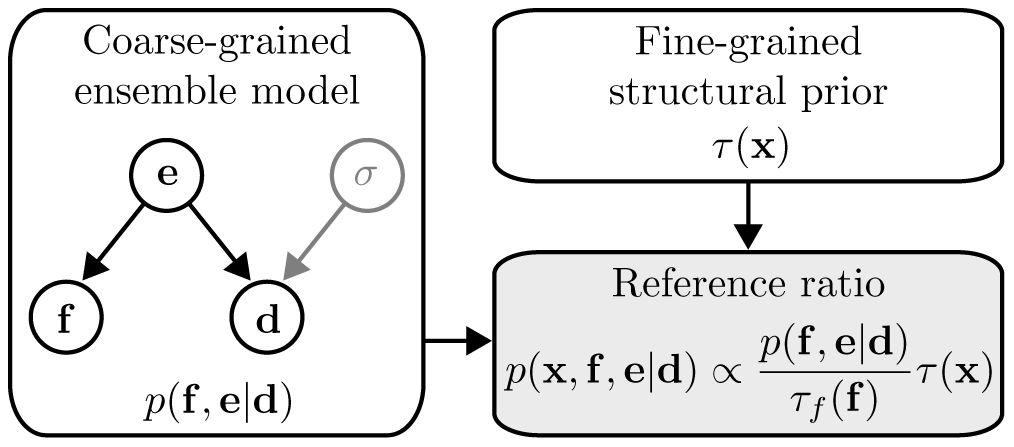
A directed graphical model of the ensemble model (on the left) and its interplay with a fine-grained conformational prior distribution (top right) through the reference ratio method, (bottom right). In the graphical model, the black circles are random variables, and the arrows determine their conditional independencies. The parameter 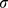, marked in grey on the left, is fixed and given, and denotes the experimental error in this particular example. 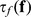 denotes the reference distribution.

We start by considering the coarse-grained representations of the distribution, 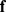, 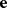 and 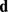, without considering the fine-grained representation, 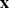. Following the Bayesian probability calculus, we formulate a posterior distribution,
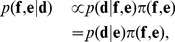
(1)where the first term is the likelihood and the second term is the prior distribution. Note that the prior of 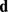 is in variant during inference and left out, hence the proportionality. The equality is due to the redundancy of 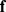 in the evaluation of the likelihood function – 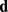 is a noisy observation of 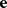, which does not involve 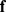. The independence assumptions of the model are shown in the corresponding graphical model in Figure 1.

Applying the product rule of probability theory to equation (1), we obtain
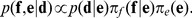
(2)

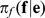
 is the prior distribution of the simulated data 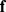 given their averaged value 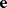, and 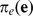 is the prior distribution over the simulated ensemble averaged data 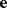.

Equation (2) is a probabilistic model of the relationship between noisy, ensemble averaged data, and conformational micro-states in a coarse-gained space. However, to obtain a probability distribution 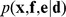 which features atomic detail, we need to combine (2) with a fine-grained physical forcefield or a probability distribution, 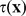. This can be done by using the *reference ratio method* [32], [33],
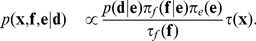
(3)

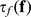
 is called the reference distribution, and is the distribution *induced* in the coarse-grained space by the fine-grained prior, 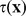. That is, the prior distribution of 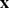, directly implies a prior distribution on 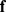, due to the parameters deterministic relationship through the forward model, 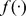. This induced prior is called the reference distribution.

The reference ratio method yields the Kullback-Leibler optimal modification of the fine-grained model 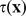 with respect to the coarse-grained information (for proof, see chapter 4 in [34]). Kullback-Leibler optimality is closely linked to the maximum entropy principle of Jaynes [35]. In essence, our approach can be seen as a maximum entropy solution given the noisy observation of an ensemble average. It should be noted that even if the distribution given by Equation (2) is unimodal, the posterior given by Equation (3) can still be multimodal due to the nature of the conformational prior, 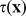.

### The relationship to other methods

The model given by Equation (3) may be reduced to the ISD framework [4]

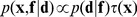
(4)


if we choose the Dirac delta function 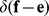 for 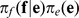 and assume that 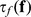 is uniform. Choosing the Dirac delta function corresponds to assuming the Boltzmann distribution is infinitely narrow. Hence, our model can be seen as a generalization of ISD. The choice of the uniform distribution for 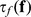 corresponds to assuming that 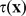 implies a suitable prior for 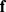 as well. This may be inappropriate in some cases (see below).

We also observe that Equation (2) may be reduced to the previously proposed maximum entropy restraining methods [27]–[29]. This is evident if we consider the case where 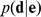 is the normal distribution and 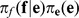 is a log-linear model 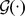 with a linear *link-function*, 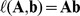. The link function allows us to include the Lagrange multipliers used to relate the coarse-grained variable 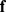 to the mean value 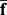 [36]. Thus, 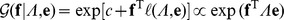. We have
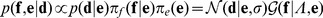
(5)where 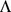 is a diagonal matrix of Lagrange multipliers. If we now consider the limit where the experimental noise vanishes we obtain,
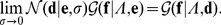
(6)


In minus 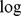-space Equation 6 is proportional to minus 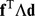. This corresponds to the empirical term of the previously reported maximum entropy method in absence of experimental uncertainty [27]. We note that this method does not explicitly account for the reference distribution 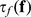 when combining the empirical term in the coarse-grained space with a fine-grained prior distribution, 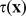. However, if the prior 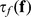 is appropriate, then the Lagrange multipliers 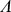 may provide the necessary means for minor adjustments.

### Reconstructing a high temperature ensemble from sparse data

To test the presented theory, we use synthetic NOE data, obtained from an ensemble of the GB1 hair-pin simulated at 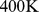 in the Profasi forcefield [30]. This simple, idealized system was chosen to minimize the chances of undersampling, as well as to avoid confounding associated with experimental data.

**Figure 2:**
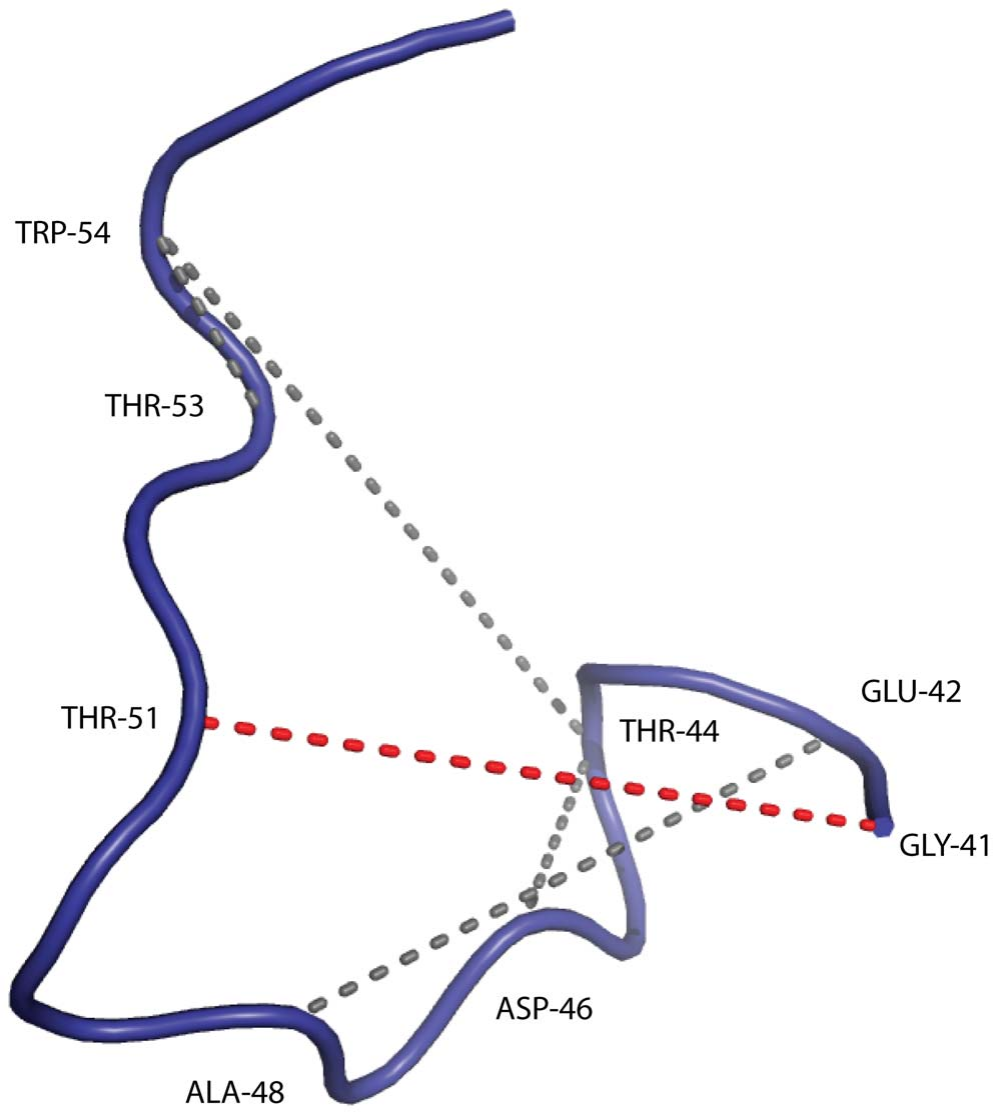
A random backbone conformation of the GB1 hairpin. The restraints listed in Table 1 are shown as dashed lines. The distance shown in red is used as the reaction coordinate 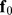 used in Figures 3 and 4. This figure was created using PyMOL (DeLano Scientific LCC).

The restraints used here are visualized on a random conformation of the GB1 hairpin in Figure 2. Historically, NOEs constitute one of the most important sources of semi-quantitative information in NMR structure determination. Under the isolated spin-pair approximation for rigid molecules, NOEs are related to an interatomic distance 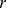 as 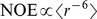 [37]. As an example, we will apply equation (2) to two cases of averaged pairwise distance data – these two cases involve the arithmetic mean 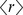, and the power-averaged mean 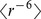, respectively. They represent two different averaging processes that are common in biophysical data.

We use the log-normal distribution as an appropriate error-model for pairwise distances derived from NOEs, which is the approach adopted by ISD [38]. The choice for the prior 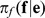 is less obvious and depends on the type of experimental data. Here, we use the exponential distribution with mean 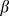, 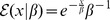, since it constitutes the least biasing continuous distribution on the positive real axis, when no higher order moments are observed [39]. The prior on 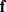 thus becomes: 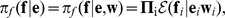 where the product runs over all data-points and the scale vector 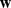 is a free parameter (discussed below). It follows that
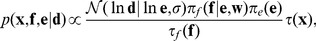
(7)where 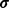 is the experimental error, which is fixed and given, and 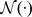 is the normal distribution. As prior on the ensemble average 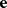 we choose 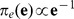. This prior has previously been shown to provide good results for variables confined to the positive real axis [40].

Equation (7) provides a general solution to the problem of modeling averaged NOE data subject to experimental uncertainty. The only parameter to be estimated is the scale vector 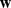, which relates 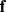 to 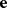 in 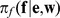.

In the ideal case, an optimal choice for 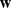 results in the desired distribution for 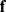 as calculated from the structures 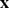. More precisely, it results in a marginal posterior distribution of Equation (7) for 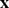, such that, when 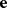 is fixed, the expectation of 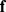 is equal to 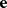. In practice, a satisfactory point estimate for 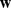 can be obtained in an iterative manner, using an empirical Bayes approach (see Materials and Methods). The parameter 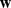 compensates for the approximate nature the reference distribution 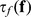, which is difficult to estimate accurately [33]. The introduction of 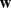 provides a simple, yet effective measure to compensate for this.

We use equation (7) to model synthetic pairwise distance restraints in the GB1 hairpin. For 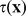 we use probabilistic models of the conformational space of the main chain [41] and the side chains [42], as these models recently yielded excellent results when combined with the ISD method [43]. As the prior distribution and the likelihood concern local and nonlocal features of protein structure, respectively, their information content shows little overlap. More informative priors, for example based on physical energy functions, can be envisaged, but this is beyond the scope of this article.

### Evaluation of inferred ensembles

**Figure 3:**
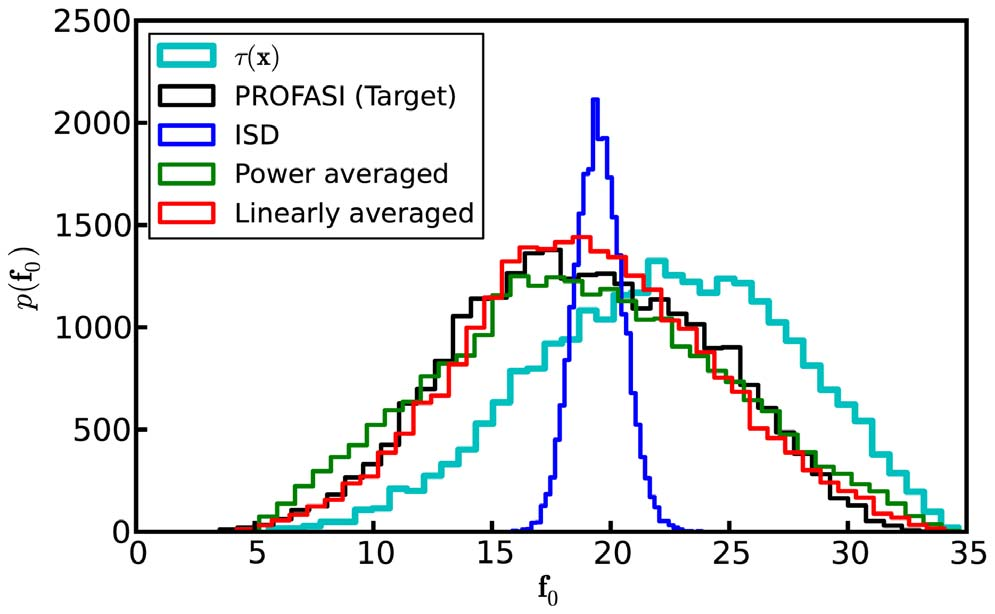
Histograms, 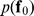, of a representative pairwise distance 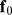 (between C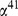-C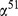, in 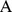) in the ensembles. The black and blue lines are obtained from the PROFASI and ISD ensembles respectively, while the cyan line represent the prior 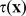. Finally, the green and red lines respectively represent ensembles obtained from the power-averaged and linearly averaged data.

**Figure 4:**
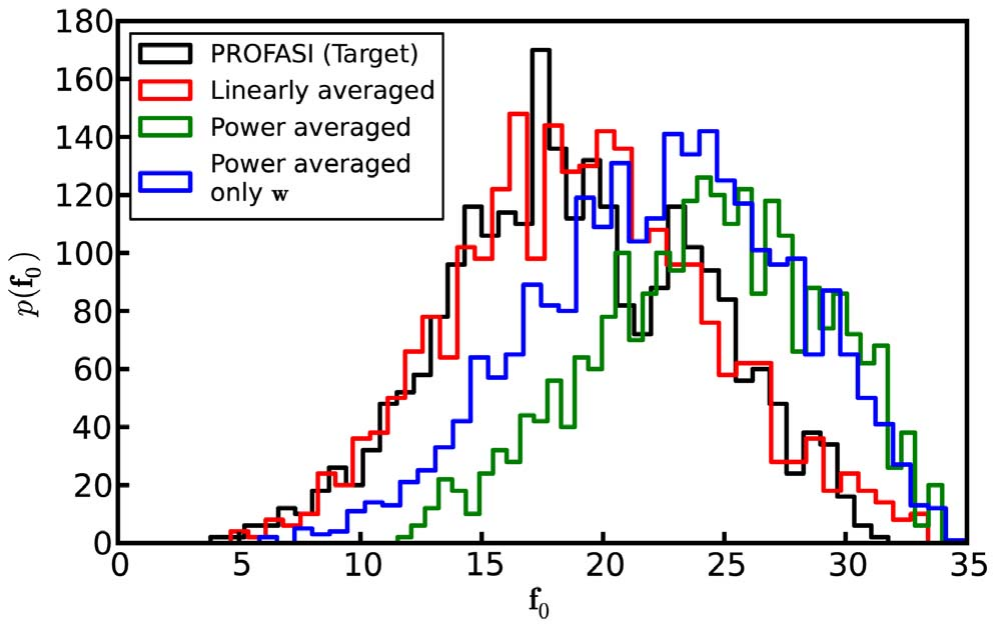
The influence of 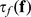 and 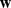 on the ensembles. The figure shows histograms, 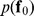, of a representative pairwise distance 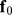 (between C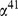-C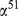, in Å ) in the ensembles obtained without the reference distribution 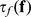 or the scale vector 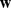. The black line denotes the PROFASI target ensemble; the red and green lines denote the ensembles obtained using the linearly and the power averaged data, respectively. The blue line denotes the case of the power averaged data without 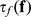, but with 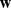.

The prior distribution used in this study concerns protein structure on a local length scale, and thus does not model long range distances accurately. Consequently, as a reaction coordinate, we chose a representative distance 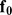 between atoms C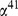 and C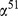 – which are separated farthest in sequence – to illustrate the long-range properties of the eight different ensembles considered here. Histograms of this pair-wise distance in the different ensembles are shown in Figures 3 and 4. This pair-wise distance is highlighted with yellow color in Figure 2.

The conformational prior and PROFASI, which was used to generate the averaged data, result in different distance distributions (Figure3). However, if we modify the prior using the reference ratio method as described above, we obtain good fits with the PROFASI distribution for both linearly and power-averaged data. The ISD ensemble, which does not take the ensemble nature of the data into account, is overly tightly peaked around the (correct) mean.

A similar pattern is observed for the distribution of the gyration radii 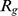. The gyration radii are not used in the estimation of the probability distributions, and can thus be used for cross-validation. The average and standard deviation of the gyration radii of the ensemble used to generate the data is 9.71±1.5Å. The ensembles obtained with our method from the power averaged and linearly averaged data resulted in a slightly higher average (10.30±1.8Å and 10.19±1.6Å, respectively), but essentially the correct standard deviation. This is an excellent result, as a perfect fit is not expected due to the sparse and noisy nature of the data. Again, the ISD ensemble provides an overly narrow distribution (9.88±0.6Å). Finally, sampling from the prior distribution alone results in an average radius of gyration of 11.34±1.8Å, which is considerably too high.

In some cases, compensating for the bias introduced by the reference distribution is not critical to obtain good results. As it constitutes an additional obstacle in terms of estimation and simulation time, we evaluate its significance on the obtained results. In the power averaged case we achieved this by chosing the reference distribution 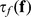 and the scale vector, 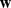, in equation (7) to be the uniform distribution and the unit vector, respectively. In the linearly averaged case, the scale vector was kept fixed equal to the 1-vector, as 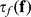 was assumed to be uniform in the results presented above. The results are shown in figure 4. In the case of the power averaged data, with 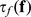 uniform and 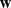 equal to the 1-vector, severely skews the distribution of the distances (green line in figure 4). When the scale vector 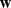 is estimated, while still assuming 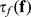 uniform, the fit improves (blue line), but without resulting in a satisfactory distribution. In the linearly averaged case, we find that a 1-vector for 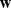 provides a good fit (red line).

If we again consider the gyration radii as providing complementary views of the ensembles, we find that the power-averaged ensemble with uniform 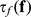 and unit scale vector yields an overly extended ensemble, 11.94±1.63Å. The results in the linearly averaged case compare to those with an estimated scale-vector, 10.34±1.69Å, presented above.

To summarize, in the power averaged case, both 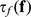 and 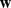 are required for a satisfactory distribution. In the case of the linearly averaged data, our results suggest that 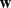 and 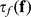 may be approximated by the 1-vector and uniform distribution, respectively. This is particularly interesting as it may be a general feature of applying other kinds of linearly averaged data. This may make the use of these types of data for restraining easier.

## Conclusions

In conclusion, we present the theoretical foundations of a Bayesian principle to infer ensembles of protein structures from noisy experimental data subject to ensemble and time averaging. We demonstrate the principle constitutes a generalization of ISD and previously proposed maximum entropy restraining approaches. Finally, the principle is successfully evaluated using synthetic experimental data of a small idealized system.

Our approach combines a coarse-grained Bayesian model of the data with a fine-grained model of protein conformational space. The combination is accomplished using the reference ratio method [33], which corresponds to a maximum entropy solution in the presence of experimental noise. The role of the reference distribution 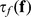 is considerable. When we assumed 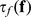 to be uniform, we were unable to construct sufficiently accurate distribution of pair-wise distance geometry, in the case of power-averaged data.

The Bayesian model may in principle be applied to denser and/or ambiguous [44] datasets and to data from other sources such as small angle **X**-ray- or neutron scattering, or other NMR experiments. Also, low-resolution data may be combined with more sophisticated physical prior distributions such as those embodied in force fields. The presented method is thus a general method to obtain physically sound ensemble models of solution and endogenous states of biomolecules, given appropriate experimental data. Practical implementation of protocols for other data sources and larger systems clearly is necessary. Possible issues arising with this methodology include insufficient sampling of the conformational space and difficult estimation procedures for reference distributions and scale parameters alike. However, the work presented herein provides the guiding principles for these future developments.

## Materials and Methods

### Synthetic datasets

A synthetic dataset was created for the GB1 hairpin (Protein data bank identifier: 1LE3; sequence variant [Y45W, F52W, V54W]). The data were generated by simulating the protein at 400K with the PROFASI forcefield [30], using Engh-Huber parameters for bond-angles and bond-lengths [45]. The high temperature was used to emulate the effect of a denatured, disordered state. A total of 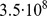 steps were performed using the Metropolis-Hastings algorithm in the PHAISTOS Markov chain Monte Carlo framework (http://www.phaistos.org). We used a Monte Carlo move set previously described [43]. Samples were saved in intervals of 5000 steps. These samples were used to form five non-redundant, averaged 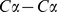 distance restraints (see Tabel 1).

To mimic the effect of distance averaging in a dipolar interaction undergoing fast motion compared to the cross-relaxation but slow motion when compared to molecular tumbling, we calculated a power averaged variant of the dataset as 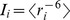, where 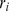 is an inter-atomic distance and the angular-brackets denote ensemble averaging [46]. We used an experimental uncertainty for the power averaged dataset 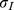 of the same relative amplitude as for the average restraint set 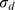, by enforcing the signal-to-noise ratio to be constant. Hence, 
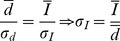
, as 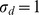, where 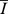 and 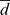 denote the datapoints corresponding to the largest average distance in the power and linearly averaged datasets, respectively. Noise with standard-deviation 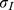 was added to the power-averaged data.

### Estimation of 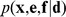 and scale vector 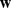


This section describes the estimation of the reference distribution 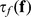 and the vector 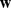 needed for the posterior distribution:
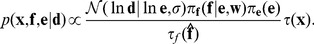
(8)


In the case of the power-averaged data, the reference distribution 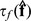 was approximated by a product of exponential distributions:
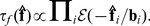
(9)


The mean 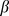 was estimated using a Monte Carlo scheme similar to that used to form the synthetic datasets, but only using the prior 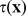, consisting of the probabilistic models TorusDBN [41] and Basilisk [42] along with a simple binary term assuring atoms do not overlap [47]. The coarse graining, 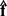, was the inverse pairwise distance between the C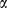 atoms listed in Table 1. For the linearly-averaged data, 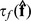 was approximated by a uniform distribution.

**Table 1:** Synthetic datasets used in this study.

C 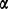 -pair	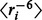	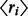
41–51	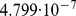	19.40
42–48	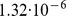	14.36
44–46	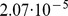	6.06
44–54	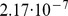	19.39
53–54	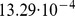	3.51

First colum: C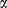 atoms involved in the pairwise distance. Second and last columns: averaged and power-averaged pairwise distances, respectively.

We obtain a point estimate of 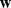 following an empirical Bayes approach. We start by initializing all the elements of 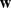 to unity. Subsequently, we sample an ensemble according to Equation (8), and update 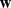 based on the sampled values of 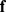 and 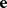. To update 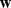 we make use of the moment estimator for the mean of the exponential distribution:
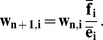


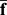
 and 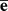 are posterior expectations of the coarse-grained variable and the ensemble averages using scale vector 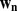, respectively, and 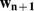 is the updated scale vector. This procedure is repeated until convergence. Convergence was assumed when fluctuations in 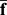 were within the experimental uncertainty. Each step in the algorithm runs for 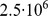 MCMC steps, and a final production ensemble is produced using 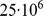 MCMC steps.

### Sampling of 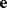


To sample 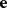 from the prior 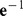 we sampled a factor 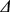 from a log-normal distribution 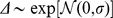, where 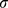 has the same order of magnitude as the experimental uncertainty. A change from 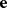 to 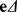 was accepted according to the Metropolis acceptance probability 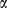:
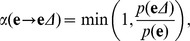



where 
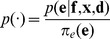
.
